# *Plasmodium falciparum* and *Plasmodium vivax* Adjust Investment in Transmission in Response to Change in Transmission Intensity: A Review of the Current State of Research

**DOI:** 10.3389/fcimb.2021.786317

**Published:** 2021-12-08

**Authors:** Colins O. Oduma, Cristian Koepfli

**Affiliations:** ^1^Department of Biochemistry and Molecular Biology, Egerton University, Nakuru, Kenya; ^2^Centre for Global Health Research, Kenya Medical Research Institute, Kisumu, Kenya; ^3^Department of Biological Sciences, University of Notre Dame, Notre Dame, IN, United States

**Keywords:** *Plasmodium falciparum* (Pf), *Plasmodium vivax* (pv), gametocyte carriage, investment in transmission, seasonality, transmission intensity

## Abstract

Malaria parasites can adjust the proportion of parasites that develop into gametocytes, and thus the probability for human-to-vector transmission, through changes in the gametocyte conversion rate. Understanding the factors that impact the commitment of malaria parasites to transmission is required to design better control interventions. *Plasmodium* spp. persist across countries with vast differences in transmission intensities, and in sites where transmission is highly seasonal. Mounting evidence shows that *Plasmodium* spp. adjusts the investment in transmission according to seasonality of vector abundance, and transmission intensity. Various techniques to determine the investment in transmission are available, i.e., short-term culture, where the conversion rate can be measured most directly, genome and transcriptome studies, quantification of mature gametocytes, and mosquito feeding assays. In sites with seasonal transmission, the proportion of gametocytes, their densities and infectivity are higher during the wet season, when vectors are plentiful. When countries with pronounced differences in transmission intensity were compared, the investment in transmission was higher when transmission was low, thus maximizing the parasite’s chances to be transmitted to mosquitoes. Increased transmissibility of residual infections after a successful reduction of malaria transmission levels need to be considered when designing intervention measures.

## Introduction

Transmission of malaria parasites from the human to the mosquito host is a crucial bottleneck in the lifecycle of the parasite. Not all *Plasmodium* species infections contribute to transmission. Infection of the mosquito depends on the presence of the sexual forms of the parasite, male and female gametocytes, in the blood, and their uptake by mosquitoes.

Over the course of the *Plasmodium* spp. intraerythrocytic 48-h cycle, only a small proportion of parasites commit to sexual differentiation and develop into gametocytes (Sinden, 1983). Once taken up by mosquitoes, male and female gametocytes fuse to form oocysts. Approximately 14 days after the uptake of gametocytes, infective sporozoites are present in the mosquito’s salivary glands (Meis et al., 1992), resulting in human infection once the next blood meal is taken.

*Plasmodium falciparum* (*P. falciparum*) and *Plasmodium vivax* (*P. vivax*) are the primary cause of malaria in humans. Both species combined present the greatest threat, accounting for the majority of malaria related morbidity and mortality. *P. falciparum* is responsible for most cases of severe clinical malaria. The duration of gametocyte development and maturation differ between *P. falciparum* and *P. vivax*. *P. falciparum* gametocyte development takes 8-12 days, during which gametocytes undergo five morphologically distinct stages (Hawking et al., 1971; Sinden et al., 1978). Immature stages (late stage I-IV) sequester in inner organs and are absent from blood circulation i.e., they sequester in inner organs until maturity (Paul et al., 2000; Eichner et al., 2001; Farfour et al., 2012). As a result of the long maturation period, gametocytes are often not present during the first wave of asexual parasitemia when clinical malaria occurs. In contrast, *P. vivax* gametocytes take only 2-3 days for maturation, and infective gametocytes are present in circulation before clinical symptoms occur (Boyd and Stratman-thomas, 1934; Boyd et al., 1936; Vallejo et al., 2016).

The switch between asexual development and sexual differentiation is one of the few times in the parasite’s lifecycle that it can adjust its strategy to maximize fitness. The parasite can prioritize either to produce more gametocytes (i.e., increase the conversion rate) to increase its chances of transmission, or increase replication of asexual parasites to enhance its survival in the host (Reece et al., 2009; Carter et al., 2013). Gametocytes that are not taken up by mosquitoes cannot return to asexual development, thus the investment in transmission will be lost.

The proportion of parasites that develop into gametocytes, i.e., the gametocyte conversion rate, differs substantially between isolates collected from patients in the field (Smalley et al., 1981; Usui et al., 2019). It might vary over the duration of untreated infections, and be adjusted in response to antimalarial treatment or other external factors (Schneider et al., 2018). Also, the duration of gametocyte circulation in peripheral blood might vary (Eichner et al., 2001), further adding to variation of transmission potential. Understanding factors (i.e., the stresses) that impact the transmission potential is critical to guide and evaluate transmission reducing interventions.

As many countries transition from malaria control to elimination, the focus shifts from diagnosis and treatment of clinical cases to understanding the full transmission reservoir, including transmission originating from subclinical infections (Bousema et al., 2014). Various interventions, e.g. vector control methods such as use of bed nets, or indoor residual spraying, and parasite screening and treatment have been key to reducing transmission. An increasing number of studies indicate that parasites adjust their investment in transmission in response to a changing environment (Mobegi et al., 2014; Gadalla et al., 2016; Ouédraogo et al., 2016; Parobek et al., 2016; Rono et al., 2018; Koepfli et al., 2021; Oduma et al., 2021). A better understanding of these processes is needed to develop novel strategies to achieve elimination. Identifying factors that result in higher investment to transmission might allow control programs to tailor their interventions towards covering these factors.

Here, we summarize the methodology to measure the commitment to transmission in natural infections, and review recent field studies assessing the impact of changes in transmission intensity across seasons or long-term (i.e., between countries differing in transmission) on the commitment to transmission and infectivity of *P. falciparum* and *P. vivax*.

## Direct and Indirect Measures of Commitment to Transmission

In recent years, much has been revealed about the molecular basis of gametocyte conversion. The underlying processes have been reviewed elsewhere (Josling and Llinás, 2015; Nilsson et al., 2015; Josling et al., 2018). In brief, the Apetala-2 transcription factor (Ap2-g) is essential for the differentiation of gametocyte-committed parasites into early gametocytes (Kafsack et al., 2014). Expression of *ap2-g* is activated by interactions of nuclear proteins *P. falciparum* gametocyte development protein 1 (GDV1) and heterochromatin protein 1 (HP1) (Eksi et al., 2012; Brancucci et al., 2014; Filarsky et al., 2018). In asexual parasites, the *Ap2-g* is epigenetically silenced (Flueck et al., 2009; Lopez-Rubio et al., 2009; Brancucci et al., 2014; Coleman et al., 2014).

In field isolates, commitment to transmission can be assessed through various methods. The most direct measure of the conversion rate can be obtained in short-term culture, i.e., by quantifying early gametocytes after 2-8 days in culture by microscopy and comparing their density to parasite density at day 0. These assays require laboratories for parasite culture to be present in the study site and are complex to perform, thus comparably few studies have adopted this method.

Studies assessing *P. falciparum* gametocyte conversion directly have found pronounced differences among isolates (Smalley et al., 1981; Poran et al., 2017; Usui et al., 2019; Prajapati et al., 2020). An early study in 1981 in Ghana found a mean gametocyte conversion rate of 7.6% among infections that already carried mature gametocytes, and of 1.3% among infections without any mature gametocytes detected by microscopy (Smalley et al., 1981). A more recent study applied similar methodology to measure the gametocyte conversion rate in 260 children presenting with clinical malaria in Ghana. The gametocyte conversion rate was determined microscopically by counting the number of early stage gametocytes at day 4 or 8 after culture and compare it to asexual parasite density at day 0. Three quarters of all isolates had detectable circulating gametocyte-committed rings. The gametocyte conversion rate varied widely, with up to 78% of all rings being committed to sexual development. The median conversion rate was low at 0.7%, and 20% of the samples had high conversion rates of >4% (Usui et al., 2019). Due to the challenges in culturing *P. vivax*, no studies have attempted to measure *P. vivax* conversion using similar methodology.

As an alternative to the direct measurement of the gametocyte conversion rate, many studies have quantified mature gametocytes in human blood samples by microscopy, or through measuring expression of gametocyte-specific mRNA transcripts by RT-qPCR, NASBA, or other molecular methods. (Koepfli and Yan, 2018; Tadesse et al., 2019). Molecular methods allow for quantification of very low-density parasites and gametocytes. Multiple molecular markers for gametocyte detection have been identified. Frequently used makers include *pfs25*, *pfs16*, *pfg377*, and *pfs230p* for *P. falciparum*, and *pvs25* (or *P. vivax*) [reviewed in (Koepfli and Yan, 2018)] Most of these genes are transcribed in the gametocyte, but translationally repressed until uptake of gametocytes by the mosquito (Mair et al., 2006; Guerreiro et al., 2014).The *Pfs25 and Pvs25* are the most widely used targets for gametocyte detection and quantification as they show limited polymorphism, are sensitive and are highly abundant in blood (Kaslow et al., 1989; Beurskens et al., 2009; Feng et al., 2011; Schneider et al., 2015). As in studies that measured conversion directly, large variation in the proportion of gametocytes compared to asexual parasites was found (Koepfli et al., 2015; Almeida et al., 2018; Tadesse et al., 2018; Kosasih et al., 2021).

While gametocyte quantification is a useful surrogate marker of infectivity, it is only an indirect measure of commitment to transmission (Koepfli and Yan, 2018). A direct comparison revealed a poor correlation between the gametocyte conversion rate and the proportion of mature gametocytes (Usui et al., 2019). Parasite densities in natural infections fluctuate across many orders of magnitude, thus differences in gametocyte densities might simply represent variation in parasite densities. Further, developing *P. falciparum* gametocytes sequester for approximately 10 days. Thus, gametocyte densities at the day of sampling might represent parasite densities up to two weeks previously. These densities are not known unless frequent follow-up sampling is conducted. *P. vivax* gametocytes develop within 2-3 days, thus gametocyte density might mirror commitment to transmission more directly.

Mosquito feeding assays are a further means to measure transmission potential. They are the gold standard to measure infectivity. Yet, as gametocyte densities, these studies measure commitment to transmission only indirectly. Infectivity can be measured as the percentage of human hosts that infect at least one mosquito, the proportion of mosquitoes infected, or the number of oocysts per infected mosquito. While gametocyte densities can serve as useful predictor for transmission potential, among individuals with gametocytes infectivity can be reduced in the presence of transmission-blocking immunity (Bousema et al., 2011), in case of an incompatibility between the parasite strain and vector species (Molina-Cruz et al., 2015; Tang et al., 2020), or if gametocytes are present, but not infective, e.g. after drug treatment (Abay, 2013), or when gametocytes are present but are not fully infective because mature gametocytes in peripheral circulation require additional 3 days to become fully infective (Smalley and Sinden, 1977; Lensen et al., 1999). While studies conducting feeding assays have overall shown a relationship between gametocyte density and the number of mosquitoes infected, infectivity varied among isolates. Even at high gametocyte densities, some blood samples did not result in all mosquitoes being infected, while some samples with very low gametocyte density were able to infect mosquitoes (Ouédraogo et al., 2016; Gonçalves et al., 2017; Bradley et al., 2018). Failure to infect mosquitos despite high gametocyte densities might be the result of transmission blocking immunity (i.e. antibodies in the human blood rendering gametocytes non-infective (Stone et al., 2018), or defense mechanisms of the mosquito (Cirimotich et al., 2010).

## Impact of Transmission Intensity on Transmission Potential

Mounting evidence suggests that malaria parasites adjust their investment in transmission in response to transmission intensity in their environment, e.g. vector abundance (Gadalla et al., 2016; Ouédraogo et al., 2016; Parobek et al., 2016; Rono et al., 2018; Vantaux et al., 2018; Koepfli et al., 2021; Oduma et al., 2021). Such adaptations are both long-term, i.e., between countries differing in transmission, and short term, i.e., across seasons.

Adjustments to differences in transmission intensity in space and time are reflected in the genome and transcriptome of malaria parasites. A study compared whole genome sequencing data from parasites from the Gambia, where transmission is low and seasonal, and Guinea, where transmission is high (Mobegi et al., 2014). *Gdv1*, which is key for early gametocyte development, stood out as one of the genes that differed most between populations (Mobegi et al., 2014). This likely represents selection for alleles adjusted to the respective transmission intensity. In Cambodia, intensified control resulted in an 80% reduction in the number of *P. falciparum* cases from 2009 to 2013. The number of *P. vivax* cases increased in the same period (Maude et al., 2014). Genomic data provided a possible clue to the reasons for this increase. *P. vivax* parasites collected over this period were sequenced, and the strongest selective sweep was found around the *ap2-g* transcription factor (Parobek et al., 2016). This suggests that *P. vivax* adjusted its investment in transmission in response to control within only a few years, resulting in higher levels of transmission despite intensified vector control.

Similar results of adaptation in response to transmission intensity were observed in gene expression studies. A study compared *P. falciparum* isolates from non-immune children with clinical malaria from three sites in East African with long-term differences in transmission intensities, i.e., Kisumu, Kenya (high), Kilifi, Kenya (medium), and Sudan (low). Expression levels of *P. falciparum ap2*-*g* differed substantially between parasites isolated from the high *versus* low transmission settings. The expression levels of *ap2-g* increased as transmission intensity decreased (Rono et al., 2018). These findings imply that in areas where malaria transmission is low*, P. falciparum* parasites invest more in transmission compared to areas where malaria transmission is high. This plasticity allows parasites in natural populations to adapt to their local environment to maintain fitness.

In another study comparing transmission potential among sites, *P. falciparum* and *P. vivax* gametocyte densities were compared in over 16,000 asymptomatic individuals in Papua New Guinea (PNG), Solomon Islands, Thailand, and Brazil (Koepfli et al., 2021). Thailand and Brazil had seen extended periods of very low transmission, potentially allowing parasite populations to adjust transmission strategies. The surveys in Papua New Guinea and Solomon Islands were conducted at times where transmission was moderate-high, or had recently been reduced. The proportion of infections with gametocytes detectable by RT-qPCR varied greatly among surveys, from 43% to 94% for *P. falciparum*, and from 23% to 78% for *P. vivax*. The proportion of gametocyte-positive infections was highest in regions with lowest transmission intensity, i.e., in Brazil, Thailand, and Solomon Islands for *P. falciparum*, and in Brazil and Thailand for *P. vivax*. In parallel, gametocyte densities and the proportion of gametocytes among all parasites tended to be higher where transmission had been low for extended periods.

**Figure 1:**
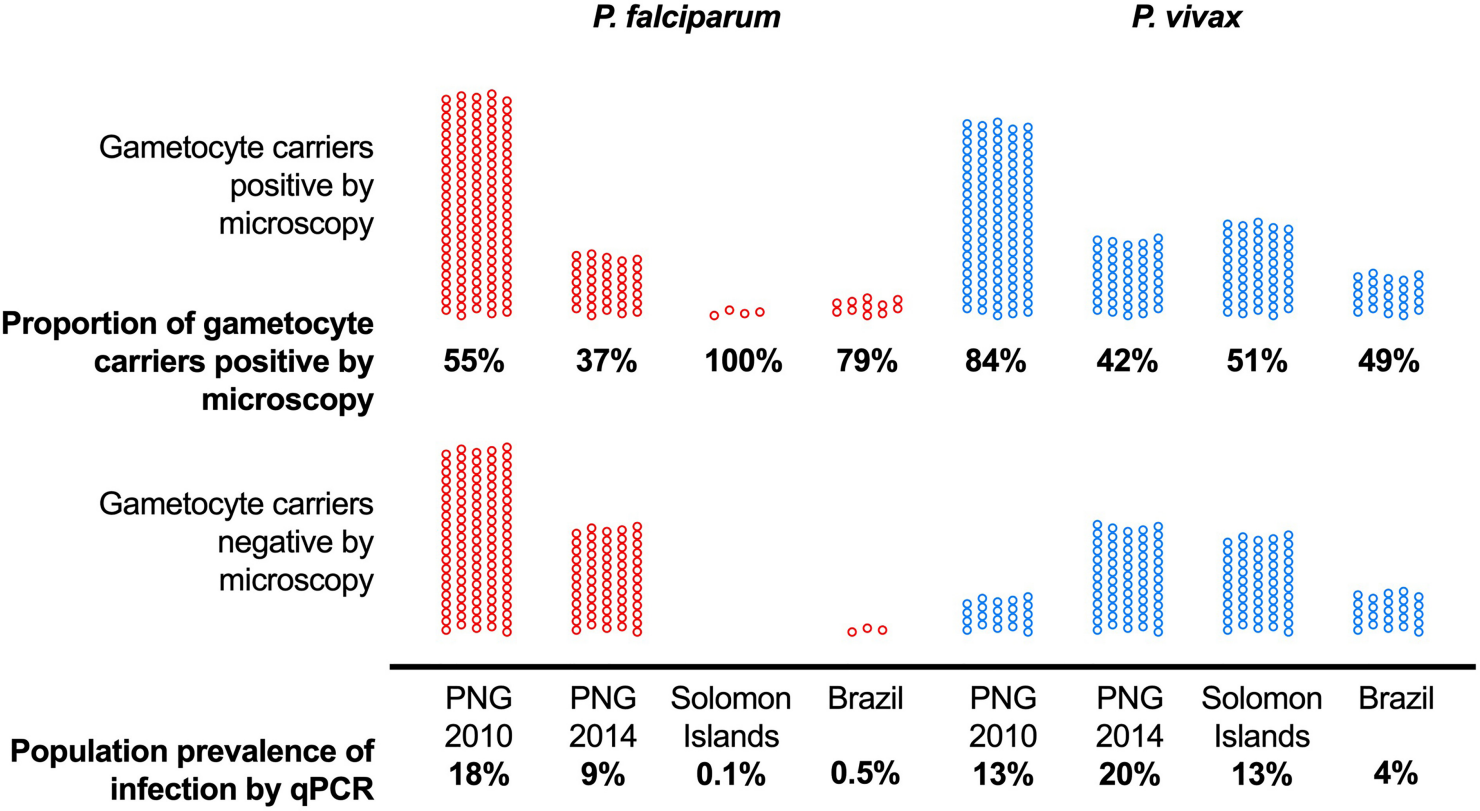
The proportion of asymptomatic *P. falciparum* and *P. vivax* infections with gametocytes (detected by RT-qPCR) that can be detected by microscopy differs among sites with different transmission intensity. Data from 10,111 surveyed individuals included. Population prevalence of infection by qPCR is indicated at the bottom and ranged from 0.1% to 18% for *P. falciparum* and 4% to 20% for *P. vivax*. Among all *P. falciparum* gametocyte carriers, 37% to 100% could be identified by microscopy, and 42% to 84% of *P. vivax* gametocyte carriers. PNG, Papua New Guinea. Data from (Waltmann et al., 2015; Koepfli et al., 2017; Almeida et al., 2018; Koepfli et al., 2021).

The combination of differences in mean *P. falciparum* and *P. vivax* parasite densities and in the investment in transmission resulted in pronounced differences in the proportion of gametocyte carriers that could be detected by microscopy (**Figure 1**). Across surveys, 37% to 100% with *P. falciparum* gametocytes detected by RT-qPCR, and 42% to 84% of *P. vivax* gametocyte carriers were positive by microscopy (**Figure 1**) (Koepfli et al., 2021). Where *P. falciparum* prevalence was very low, i.e., in Brazil and Solomon Islands, most gametocyte carriers were positive by microscopy. These infections could thus be diagnosed by mass screen and treat programs (Koepfli et al., 2021).

The comparison of results from mosquito feeding assays across several sites also pointed adaptations to transmission intensity. Across three sites differing in *P. falciparum* transmission intensity, 1209 feeding experiments were conducted. A total of 39 individuals infected at least one mosquito. In Burkina Faso, where transmission was highest, almost all (25/27) individuals that could infect mosquitoes were positive for asexual parasites or gametocytes by research-grade microscopy. In Kilifi and Mbita, Kenya, where transmission is lower, 1/3 and 2/9 P*. falciparum* infectious individuals were submicroscopic. While these numbers are low and the differences do not reach statistical significance, the higher infectivity of submicroscopic infections in low-transmission settings might point to a higher proportion of gametocytes among all parasites, and thus might indicate a higher gametocyte conversion rate (Gonçalves et al., 2017).

## Impact of Seasonality on Transmission Potential

In many malaria-endemic countries transmission occurs primarily during the wet season, when vectors are plentiful. Increasing the investment in gametocytes in the transmission season offers optimal fitness to the parasite population as it maximizes chances of onward transmission. Indeed, changes in the commitment to transmission were found across seasons in countries where malaria transmission is seasonal.

**Figure 2:**
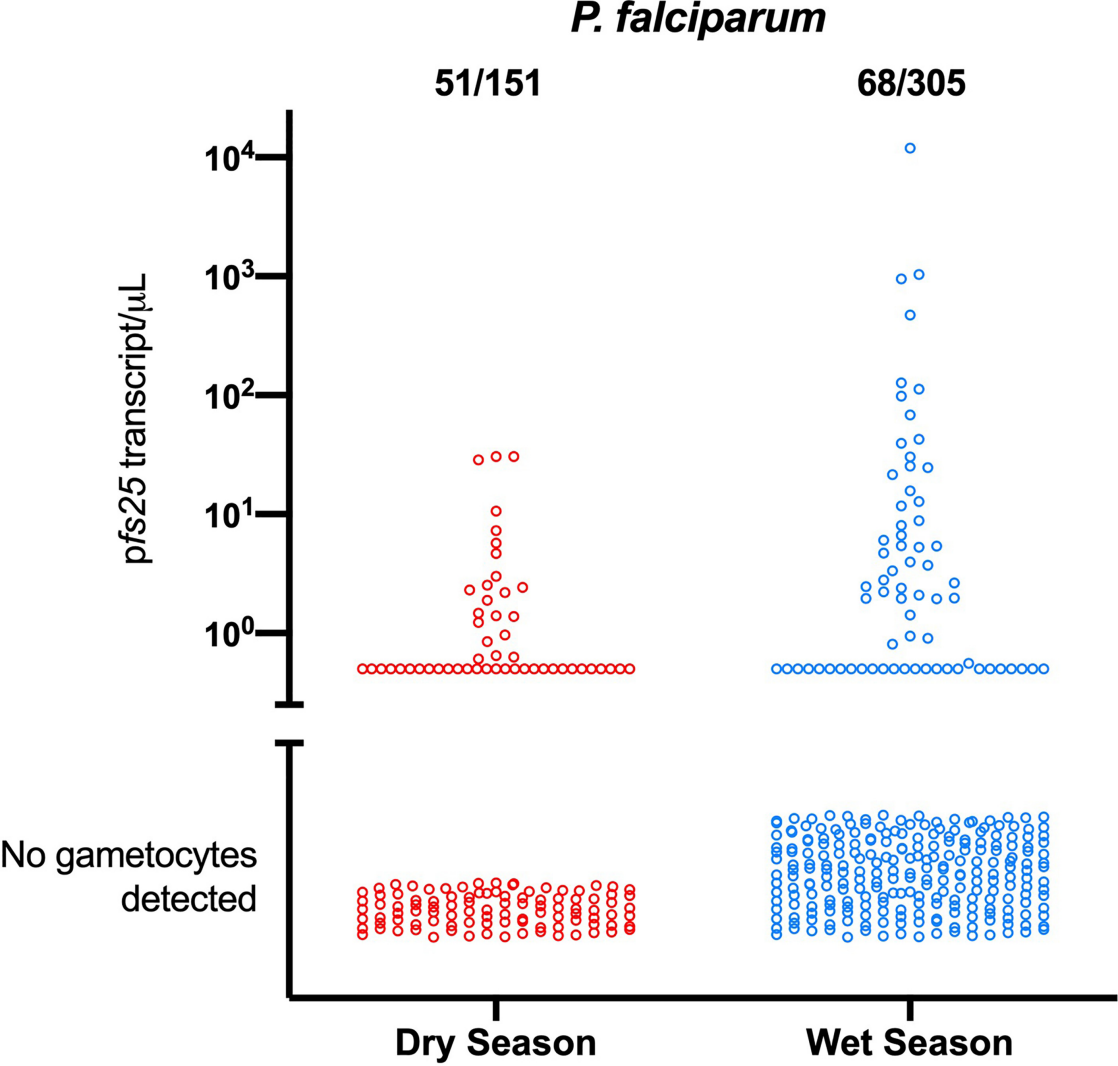
Differences in the proportion of *P. falciparum* gametocyte-positive infections and gametocyte densities between the dry and the wet season in western Kenya (Oduma et al., 2021). In the wet season, fewer infections carried gametocytes, yet mean gametocyte densities were over 3-fold higher. Mean parasite densities did not change, indicating an increased investment in transmission.

Two studies have compared *P. falciparum* gametocyte densities and parasite densities in the wet and dry seasons. In western Kenya, a total of nearly 3000 individuals were sampled in the wet and the dry seasons to screen for *P. falciparum*. Infections were diagnosed by qPCR, and gametocytes quantified by RT-qPCR. Prevalence of infection differed only moderately between seasons, it increased from 13.5% in the dry season to 17.5% in the wet season. Mean parasite density was below 10 parasites/µL, and did not differ between seasons. A lower proportion of infections carried gametocytes in the wet season, but *pfs25* transcript densities were over 3-fold higher (**Figure 2**) (Oduma et al., 2021). In the dry season, very few individuals carried gametocytes that likely could infect mosquitoes. In the wet season, this number was much higher. The increase in gametocyte density while asexual parasite density changed little reflects an increase in the proportion of gametocytes among all parasites, and thus a possible indication of an increased investment in transmission.

A similar result was found in Sudan, where *P. falciparum* gametocytes densities were measured by RT-qPCR in 25 individuals that sustained chronic, asymptomatic infections across two transmission seasons. Gametocyte densities were higher in the period just before the main transmission season (i.e., when *Anopheles* mosquitoes are present but no clinical cases are reported), compared to the preceding transmission-free season (i.e., when neither vectors are present nor clinical cases are reported). As in the study in Kenya, *P. falciparum* parasite density did not change between the two seasons, suggesting an increase in the gametocyte conversion rate (Gadalla et al., 2016).

Several studies compared mosquito infectivity from *P. falciparum* asymptomatic carriers between seasons. In Burkina Faso, 130 individuals were randomly selected and gametocytes and infectivity quantified in the dry season, the beginning of the wet season, and the peak of the wet season. *P. falciparum* Parasite prevalence (84%-94% by molecular diagnosis) and gametocyte prevalence (60%-68% by molecular diagnosis) did not differ substantially across time points. In contrast, infectivity to mosquitoes, as assessed in membrane feeding assays, varied greatly. Only 15% of individuals were infective in the dry season, compared to 48% at the beginning of the wet season and 34% at the peak of the wet season (Ouédraogo et al., 2016).

To understand the patterns in infectivity across seasons in areas of varied transmission intensity, community surveys involving 1216 observations were carried out in regions of varied transmission intensities, Burkina Faso (high), Mbita, Kenya (moderate) and Kilifi, Kenya (low) to determine the infectiousness of mosquitoes across wet and dry seasons. *P. falciparum* parasite positivity by qPCR for Burkina Faso wet vs dry was 83.6%, 50.0%, and Kilifi wet vs dry was 37.4% and 38.4%, and by microscopy for Mbita wet vs dry was 25.7% vs 28.2%. Across Burkina Faso and Kilifi sites, *P. falciparum* gametocyte carriers and densities by molecular methods were significantly higher in the wet season. To assess the infectivity of study participants, 1209 mosquito feeding assays were performed. A total of 39/1209 (3.2%) of individuals infected at least one mosquito. Proportion of mosquitoes infected per season across the sites did not follow a pattern i.e., Kilifi; dry 0/3046 (0%) vs wet 4/7716 (0.1%), Mbita; dry 28/7071 (0.4%) vs wet 5/6842 (0.1%), Burkina Faso; dry 110/17231 (0.6%), vs wet 121/7749 (1.6%) (Gonçalves et al., 2017).

In Cambodia, asymptomatic individuals were followed in dry and wet seasons to determine infectivity to *An. dirus* mosquito. The study involved 32 and 29 participants for dry and wet seasons respectively. *P. falciparum* gametocyte positivity by RT-PCR was higher in dry season whereas infectivity was relatively high in rainy season. Gametocyte positivity was 71.7% (43/60) and 49% (27/55) among *P. falciparum* infections in the dry and wet seasons respectively. Infectivity among fed *An. dirus* mosquitoes were 2.2% (2/91) and 3.6% (6/168) in dry and rainy seasons respectively (Vantaux et al., 2018).

## Concluding Remarks

An increasing body of research measuring the gametocyte conversion rate, gametocyte densities, and infectivity indicates that *P. falciparum* and *P. vivax* are able to adapt their investment in transmission in response to seasonality and transmission intensity in a site.

Across seasons, *Plasmodium falciparum* is able to increase gametocyte densities and infectivity when transmission increases and vectors are present. This strategy prevents the parasite from spending resources on gametocytes production when the chances of onward transmission is low. Likely, it also benefits the parasite as little or no natural immunity to sexual stages is acquired during the transmission-free season (Kengne-Ouafo et al., 2019). Naturally acquired immunity against gametocytes reduces infectivity. Such immunity is likely short-lived, i.e. the result of recent exposure rather than cumulative exposure (Ouédraogo et al., 2011). Absence of acquired immunity at the start of the transmission season likely results in higher infection success in mosquitos. The stimuli that causes this adjustment is poorly understood. *P. falciparum* parasitized red blood cell - derived microvesicles promote sexual differentiation (Mantel et al., 2013; Regev-Rudzki et al., 2013), however little is known about sensing and signaling pathways and factors that trigger this process. Similarly, there is evidence that the density of uninfected mosquito bites increases in at the start of transmission season (Paul et al., 2004). However, it is not known whether parasites sense abundance of the uninfected mosquito bites at the start of transmission season or they might sense physiological factors of the host body that change in response to seasonality. Increase in transmissibility in the wet season strengthens the rationale for control activities that are adjusted to seasonality, such as seasonal chemoprevention (Konaté et al., 2020; Tchouatieu et al., 2020).

An opposite effect is observed when transmission levels decrease across multiple years, or when parasite population are compared between sites of high and low transmission. The proportion of gametocytes among all *P. falciparum* and *P. vivax* parasites, and infectivity increase when transmission is lower. These long-term changes, which are reflected in the genome, increases the *Plasmodium* parasite’s chances for onward transmission even when few vectors are present.

Numerous questions on stimuli affecting the gametocyte conversion rate remain to be answered. Evidence whether the conversion rate is influenced by asexual parasitemia is conflicting. While it was observed in some field and laboratory studies (Carter and Miller, 1979; Schneider et al., 2018; Usui et al., 2019), it was not seen in large population based surveys (Koepfli et al., 2021). It is not known whether presence of mature gametocytes impact on conversion rate. Little is known about the gametocyte epidemiology of other *Plasmodium* sp. infecting humans, i.e., *P. malariae*, *P. ovale*, and *P. knowlesi*, which is emerging in parts of south-east Asia. It remains to be shown whether coinfection of any of these species with *P. falciparum* results in altered gametocyte densities of either species.

Few studies have been conducted on *P. vivax* transmission epidemiology. It is not clear whether observed differences between *P. falciparum* and *P. vivax*, such as different responses to malaria control efforts on Cambodia (Maude et al., 2014), point to different transmission strategies of the two species. A hallmark of *P. vivax* biology is the formation of dormant liver stages that can result in relapsing blood-stage infections weeks to months after the initial infection (Markus, 2012). Relapses result in gametocytemia and thus renewed possibilities for onward transmission (Wampfler et al., 2017). An analysis of historical *P. vivax* data from Finland indicated that *P. vivax* relapses might be triggered by mosquito bites, and thus presence of gametocytes and vectors coincides (Huldén et al., 2008). In how far *P. vivax* is able to time the occurrence of relapses to increase transmission success, and whether such a process is employed as alternative to adjusting the gametocyte conversion rate, is not known.

Increased infectivity of residual infections in populations where transmission has been greatly reduced might be a threat to malaria elimination. Higher infectivity of subpatent infections will reduce the effectiveness of control activities such as reactive case detection (Stuck et al., 2020) or mass screen and treat (Kosasih et al., 2021), which typically rely on rapid diagnostic test or microscopy for diagnosis. This process might be balanced by a higher proportion of all gametocyte carriers being microscopy positive in very low transmission settings (Koepfli et al., 2021). In conclusion, understanding the investment in transmission of parasite populations in different settings can help in informing the design of effective malaria control and elimination strategies.

## Author Contributions

COO and CK performed the search for relevant literature, read manuscripts, and wrote the final manuscript. All authors contributed to the article and approved the submitted version.

## Conflict of Interest

The authors declare that the research was conducted in the absence of any commercial or financial relationships that could be construed as a potential conflict of interest.

## Publisher’s Note

All claims expressed in this article are solely those of the authors and do not necessarily represent those of their affiliated organizations, or those of the publisher, the editors and the reviewers. Any product that may be evaluated in this article, or claim that may be made by its manufacturer, is not guaranteed or endorsed by the publisher.
